# Blood Type Associated with the Risk of COVID-19 Infection in Pregnant Women

**DOI:** 10.3390/diagnostics13071338

**Published:** 2023-04-04

**Authors:** Rosalba Sevilla-Montoya, Addy C. Helguera-Reppeto, Irma E. Monroy-Muñoz, Tania A. Vargas-Pavia, Elías I. Valdés-Montoya, Mario Solis-Paredes, Johnatan Torres-Torres, Rafael Velazquez-Cruz, José Esteban Muñoz-Medina, Claudia Martinez-Cordero, Alberto Hidalgo-Bravo

**Affiliations:** 1 Reproductive Research and Perinatal Health Department, National Institute of Perinatology, Mexico City 11000, Mexico; 2 Immunobiochemistry Department, National Institute of Perinatology, Mexico City 11000, Mexico; 3 Laboratory of Bone Metabolism, National Institute of Genomic Medicine (INMEGEN), Mexico City 14610, Mexico; 4 Surveillance and Epidemiological Research Laboratories Division, Mexican Institute of Social Security, Mexico City 06700, Mexico; 5 Regional Hospital of High Specialty of the Bajio, Guanajuato 37660, Mexico; 6 Genomics Medicine Department, National Institute of Rehabilitation, Mexico City 14389, Mexico

**Keywords:** blood type, COVID-19, pregnancy, risk factor, SARS-CoV-2

## Abstract

COVID-19 forced us to investigate risk factors to provide the best medical attention, especially in vulnerable groups, such as pregnant patients. Studies in other populations have analyzed blood groups in relation to infection, complications, and death. The present study aimed to analyze the association of blood groups with the risk of infection and complications in pregnant women and newborns from the Mexican-Mestizo population. We studied 1906 individuals. Quantitative variables were analyzed through the Student’s *t*-test. Categorical variables were analyzed through Pearson’s chi-square test, and logistic regression was used to analyze the association between categorical variables and outcomes. No significant association was observed between blood groups and infection risk. Individuals with the AB blood type are at higher risk for developing severe disease, although blood groups do not seem to be involved in the risk of SARS-CoV-2 infection. However, the AB blood group could be considered a risk factor for developing severe COVID-19 in the Mexican population.

## 1. Introduction

Pandemics, such as the one we have been living with since 2019, appear periodically in human evolution. The preparation needed to prevent a major impact of these diseases indicates a close analysis of its details, especially in vulnerable groups, such as pregnant women [1]. Pregnancy is considered an adaptation challenge for all organisms. Its inherent physiological changes increase the risk of morbidity-associated infectious diseases, such as the SARS-CoV-2 infection. An immunocompromised state, an alteration of the pulmonary function, and a hypercoagulable estate are directly implicated in the augmented risk [2]. Several risk factors, including age, gender, ethnicity, smoking, hypertension, diabetes, and chronic cardiovascular diseases, have been investigated to identify the population at the highest risk of SARS-CoV-2 infection and disease severity. ABO blood-type antigens have been extensively studied as protection or predisposition factors for infectious or non-infectious diseases. The precise mechanism underlying their effect on human disease has not been fully elucidated [3]. Current evidence suggests that blood antigens play a role in protection against diseases such as malaria and diabetes. On the other hand, they can also function as risk factors, predisposing individuals to diseases such as heart conditions, cancer, and infectious disease [4]. Moreover, existing data suggest that individuals with blood group AB have an increased risk of cognitive impairment, hypertension, obesity, dyslipidemia, cardiovascular disease (CVD), and diabetes, independent of geographic region, age, ethnicity, and gender [3]. Blood groups can also be related to infectious diseases; for example, blood group O has been associated with an increased incidence of cholera and tuberculosis, whereas blood group AB is associated with a higher incidence of *E. coli* and *Salmonella* [3]. 

Recently, the association between blood type and COVID-19 infection and disease severity has been deeply analyzed [5,6]. Some research groups have tried to establish an association between blood type and the outcome of patients presenting with COVID-19. They determined that blood type was not associated with the risk of intubation or death. Nevertheless, they suggested a relationship with the risk of having a positive test in the population from the United States [5]. In the UK, a population of pregnant women, including different ethnic groups, was studied. Their data suggested that the risk for COVID-19 infection was higher in individuals carrying blood group A and lower in subjects with blood group O [7]. Pregnancy may increase the risks of COVID-19 complications, particularly thrombotic events. Analysis of specific risk factors could help us to categorize patients at major risk of suffering complications [8].

Different mechanisms could be involved in the effect of the ABO blood antigens on the response to the SARS-CoV-2 infection. For example, individuals with blood group O have anti-A and anti-B antibodies, and SARS-CoV-2 can replicate in epithelial cells expressing A and B antigens in the lungs. Anti-A and anti-B antibodies can target the infected lung epithelial cells conferring protection to these individuals [7]. Another possible mechanism is related to thrombus formation. Thrombotic events seem to be involved in the morbidity and mortality caused by the SARS-CoV-2 infection. Some reports indicate that blood group O carriers have lower biological activity and a circulating concentration of the von Willebrand factor. This protein acts as a carrier of the coagulation VIII factor, leading to lower risks of thromboembolic diseases [9]. Meanwhile, some hypothesis has emerged explaining that SARS-CoV-2 may carry ABO(H)-like structures in its envelope glycoproteins and could be transmitted due to a protective effect of the ABO antibodies. ABH antigens could allow easier access of SARS-CoV-2 to the host´s cells, thereby increasing their risk of suffering thromboembolic events [10]. 

The distribution of blood groups in the Mexican population has been previously described by Canizales et al. They found the distribution as follows: O: 61.82%, A: 27.44%, B: 8.93%, and AB: 1.81%. In this same analysis, the observed distribution of the Rh group was as follows: Rh+: 95.58% and Rh−: 4.42% [11]. The specific risk of testing positive, having a COVID-19 infection, or having a severe disease associated with blood type has not been determined in our population, especially in pregnant women, a vulnerable group in this pandemic. The National Institute of Perinatology in Mexico City is one of the centers attending pregnant women infected with SARS-CoV-2. All patients attending the institute were tested for COVID-19. This scenario allowed us to diagnose SARS-CoV-2 infection even in asymptomatic patients. The General Hospital of Mexico, another tertiary-level center, gathers symptomatic patients with severe diseases. 

In some countries, such as Mexico, COVID-19 has been a leading cause of death in pregnant women, with a maternal mortality ratio increased by 56.8% after the first year [12]. Although only hypotheses have emerged from this knowledge, it is important to recognize the risk factors that might unravel the group of women requiring closer vigilance from clinicians. This identification of the women at higher risk would help to prioritize the management and treatment of women affected by this disease. 

This study aimed to look for an association between blood groups A, B, AB, and O and the possibility of having COVID-19, severe disease, and maternal death in populations attending two of the reference centers in Mexico City.

## 2. Materials and Methods

### 2.1. Study Population

The study population was recruited at the National Institute of Perinatology (INPer) in Mexico City and the General Hospital of Mexico (HGM) from April 2019 to October 2020. During this period, a SARS-CoV-2 test using qPCR was performed on all pregnant women attending the INPer and HGM needing medical attention, obstetric ultrasound, hospitalization, or an invasive procedure. We also included the children born during this period to the pregnant women studied. The participants were randomly selected; a total of 1906 individuals with a conclusive result of the SARS-CoV-2 test were included in the analysis. From the included study population, 1330 individuals had a negative test against SARS-CoV-2, and 576 participants had a positive test. Among the positive mothers, 113 presented severe disease. Severe disease was defined according to the classification described in the COVID-19 Treatment Guidelines published by the NIH. This classification describes the clinical spectrum of the disease. It defines a severe disease as individuals who have SpO_2_ < 94% on room air at sea level, a ratio of arterial partial pressure of oxygen to fraction of inspired oxygen (PaO_2_/FiO_2_) < 300 mm Hg, a respiratory rate > 30 breaths/min, or lung infiltrates > 50%.

All protocol procedures were according to the declaration of Helsinki of 1964 and further amendments. In addition, the research protocol was approved by the institutional ethics committees from both institutions (approval number 2020-1-32, date approved January 2020). All adult participants signed an informed consent form, individuals younger than 18 signed an assent form, and their legal guardians signed an informed consent form. In the case of newborns, their parents signed the informed consent form. 

### 2.2. SARS-CoV2 Detection

For SARS-CoV-2 detection, nasopharyngeal swabs were collected for adult participants. In the case of newborns, oral and rectal swabs were obtained at birth. RNA isolation from maternal and newborn swabs was carried out using the Quick-RNA-Viral Kit (ZYMO Research, Irvine, CA, USA), according to the manufacturer’s instructions.

SARS-CoV-2 was detected through RT–PCR according to the Berlin Protocol described at the Drosten’s Laboratory from the Institute of Virology at the Medicine Berlin University. This protocol amplifies the *RdRP* and *E* viral genes, and the *RNaseP* human gene was also amplified as an internal control. For symptomatic women with a negative result, a second test using the CDC protocol was performed. The CDC protocol amplifies three fragments of the N viral gene and the *RNaseP* human gene as a control. All RT–PCRs were run in triplicate on a StepOne Plus system (Thermo Fisher Scientific, Waltham, MA, USA). A test was considered inconclusive when the human gene did not amplify. 

### 2.3. Statistical Analysis 

Quantitative variables are presented as means and standard deviation (SD). A Student’s *t*-test was used to compare quantitative variables. Categorical variables are presented as proportions. Pearson’s chi-square test was used for proportion comparison. A logistic regression model was used to analyze the association between outcomes and categorical variables. The outcomes considered were a positive test for SARS-CoV-2, severe disease, and death. For some comparisons, participants with different blood types were grouped together. For example, non-O individuals included blood types A, B, and AB. Non-A individuals included blood types O and B. A *p*-value < 0.05 was considered statistically significant. All statistical analyses were performed using the Statistical Package for the Social Sciences (SPSS) version 25.

## 3. Results

### 3.1. Distribution of Blood Groups across the Study Population

**Figure 1:**
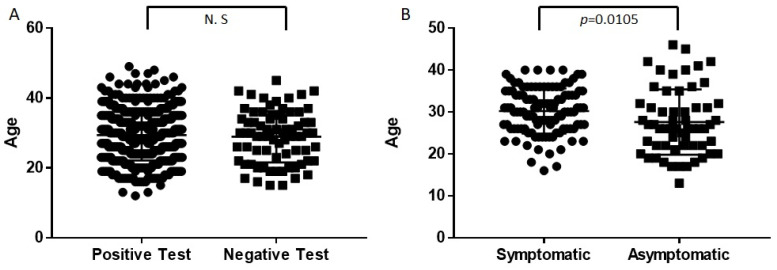
Age comparison. (**A**) Comparison of age between individuals with a positive test for SARS-CoV-2 versus individuals with a negative test revealed no significant differences. (**B**) Mothers with a positive SARS-CoV-2 test were divided into symptomatic and asymptomatic; age comparison between them revealed that symptomatic women were older than asymptomatic (*p* = 0.0105). Mean comparison was performed using the Student’s *t*-test.

A total of 1906 individuals were included in the present analysis, including 1526 pregnant women and 380 newborns. Table 1 depicts the distribution of the study population regarding the SARS-CoV-2 test result divided by blood group. Blood group percentages in the total population are also presented. The distribution of the blood groups in the total study population agrees with the epidemiological data defined for the Mexican population. The O group was the most frequent, followed by the less frequent A, B, and AB. The distribution of the RH group in the entire study population was as follows: 91.7% were Rh+, and 2.6% were Rh-. The mean age of the mothers with a positive SARS-CoV-2 test was 29.58 (SD 7.67) years, while the mean age of the mothers with a negative SARS-CoV-2 test was 28.95 (SD 7.38) years. By performing a Student’s *t*-test, we did not observe statistically significant differences between means of positive versus negative women (*p* = 0.502). Among the mothers with a positive SARS-CoV-2 test, the mean age of the asymptomatic women was 27.61 years (SD 7.80), while the mean age of the women with severe COVID-19 was 30.23 years (SD 5.36). Through a Student’s *t*-test, we identified a statistically significant difference in the mean age (*p* = 0.0105) (Figure 1). 

### 3.2. Association of Blood Groups with COVID-19 

To investigate the possible role of the ABO blood groups in COVID-19, we first analyzed if there was an association between blood groups and the risk of having a positive SARS-CoV-2 test. Logistic regression analysis did not show a significant association of the blood types with a positive result (Table 2). We also compared the proportion of each blood group between the positive and negative individuals; however, the result remained insignificant (*p* = 0.800). Afterwards, we investigated whether the risk could be different between mothers and newborns. The association remained non-significant even when mothers and newborns were analyzed as separate groups (*p* = 0.743 and *p* = 0.945, respectively). Previous reports in other populations have suggested that the A blood group is a risk factor for suffering the SARS-CoV-2 infection. Therefore, we intentionally grouped individuals into two groups, carriers of A alleles and non-carriers of A alleles. Comparison between these two groups did not show a statistically significant association (*p* = 0.756, OR 1.038 CI 0.820–1.315). Proportion analysis between these two groups was also not significant (*p* = 0.756). We further divided these two groups into mothers and newborns as independent groups; nevertheless, the association remained non-significant (*p* = 0.843 and *p* = 0.766, respectively). In addition, in some populations, blood group O has been suggested as a protective factor against the SARS-CoV-2 infection. Therefore, we divided the study population into individuals with O Blood group and non-O blood group. The O blood group did not have a significant association with the risk of infection *(p* = 0.691, OR 1.045 CI 0.842–1.295). We also made a further division of these two groups into mothers and newborns. The association remained non-significant after analyzing mothers and newborns as separate groups (*p* = 0.939 and *p* = 0.945, respectively). We further analyzed whether the Rh factor could confer an augmented risk of having a positive test. Comparison of Rh+ versus Rh- individuals did not reveal a significant association (*p* = 0.936, OR 1.025 CI 0.554–1.898). We also divided the study population into mothers and newborns to analyze the effect of the Rh factor. Nevertheless, no significant associations were observed (*p* = 0.717 and *p* = 0.350, respectively). 

Afterwards, we analyzed the patients with severe COVID-19 as an independent group. Severe symptoms were observed in 113 mothers and none of the newborns. Among the mothers with severe disease, we observed five deaths. First, we investigated whether there could be an association between the presence of severe disease and blood groups. We observed a higher frequency of individuals with severe disease in the AB blood group than in other blood groups (*p* = 0.029, Table 3). We also analyzed if the Rh factor was involved in developing severe disease. Nonetheless, there was no statistically significant difference when comparing Rh+ versus Rh- women (*p* = 0.645, OR 0.695 CI 0.148–3.267). 

Among the mothers with severe disease, five died due to respiratory insufficiency related to thrombotic complications, representing 4.4% of women with severe disease. We looked for an association of maternal death with blood groups among women who presented with severe disease. However, the risk of maternal death was not associated with the blood group (Table 4). We also analyzed if there was an association between the occurrence of death and the Rh factor. Nevertheless, the result was not statistically significant (*p* = 0.998). 

## 4. Discussion

The COVID-19 pandemic has caused more than 660 million cases and 6.6 million deaths worldwide [13]. Although vaccination has changed the clinical course of the disease, in most cases, establishing infection risk factors may help us to determine the population at higher risk. Identification of the population at higher risk is the cornerstone for designing health policies for a better distribution of human and economic resources. One of the most widely available and inexpensive laboratory tests is determining ABO blood type. Recent evidence unravels the importance of determining risk factors, such as blood type, for the prioritization of individuals at higher risk of having COVID-19, severe disease, and death.

Blood groups are defined by ABO alleles and their antibodies [14]. Antigens derived from the ABO blood groups can be found in erythrocytes, endothelial vascular cells, and respiratory and digestive tracts [15]. Previous data have demonstrated a difference in the host susceptibility to infectious agents depending on blood group antigens, which can be used as receptors or co-receptors by different microorganisms [14]. Regarding the role of ABO blood groups in COVID-19, numerous research groups have studied different populations [6,16]. Kabrah et al. determined, through a systematic review and meta-analysis, that SARS-CoV-2 infects ABO blood groups at different rates, with A and O individuals being the most affected. It is important to remember that this analysis was not focused on pregnant women and did not include the Mexican population [6]. Some other studies carried out on the general population have reported conflicting data [17,18]. A meta-analysis revealed that blood type O was related to a decreased risk of COVID-19 infection compared to non-O blood types (OR: 0.699, 95% CI: 0.635–0.770, *p* < 0.001). It also showed that individuals with blood group AB were related to an increased risk of severe COVID-19 compared to blood group non-AB with an OR of 2.424 (95% CI: 0.934–6.294, *p* = 0.069). This meta-analysis did not report significant associations related to death. This last study included mainly the Chinese population [19]. 

A few studies have considered the inclusion of pregnant women within the population study. A large study carried out on the North American population included 2648 pregnant women. Nevertheless, the authors compared the risk of infection between pregnant and non-pregnant women and found a reduced risk of infection in the group of pregnant women. They performed an analysis concerning blood group as a risk factor for infection. They did not observe differences between groups, though they did not analyze pregnant women as an independent group [20]. Another study on the Croatian population included pregnant women in the group of SARS-CoV-2-positive patients, although the analysis performed in this work did not analyze pregnant women as an independent group. Furthermore, the control group consisted of blood donors, i.e., no pregnant women were included in the control group [21]. One study analyzing exclusively pregnant women from Turkey observed a higher frequency of the O blood group in women who developed severe COVID. However, this study should be taken cautiously since the number of women included was relatively small [22]. In a study conducted in Spain, including exclusively pregnant women, the proportion of asymptomatic women was significantly higher in non-O groups when compared with the O group. Nevertheless, no disease severity between groups was observed in symptomatic women [23]. Another study carried out in Romania included exclusively pregnant women. The authors compared the risk of having a positive SARS-CoV-2 test between blood groups, and they also considered the Rh factor. They did not find a significant association; nevertheless, their group of pregnant women included less than 50 individuals [24]. 

Only one previous study analyzed the relationship between blood groups and SARS-CoV-2 infection and clinical outcomes in Mexican pregnant women. In this previous study, the authors analyzed 100 positive women and 100 negative women for SARS-CoV-2. They did not observe a significant association between blood groups and susceptibility to SARS-CoV-2 infection or fatal outcomes [25]. In the current study, we did not find significant differences regarding infection rates between ABO or Rh blood groups. However, we observed that pregnant women with the AB blood group showed an increased risk of developing severe COVID-19. These data correlate with a meta-analysis performed in 2020, including mainly the Chinese population, where an increased risk of severe COVID-19 was found in carriers of the group AB [19]. These findings could be related to the structural and configurational similarities between the A antigen and parts of the angiotensin-converting enzyme 2 (ACE2) receptor, leading to higher cell adhesion of SARS-CoV-2 [26]. Furthermore, Abdelmassih et al. proposed that furin levels might be reduced in blood type O individuals based on a previous report of a negative relationship between blood type O and furin-related protein convertases. This could lead to the assumption that individuals with non-O blood type might have higher levels of furin, which could increase SARS-CoV-2 cell entry mediated by the preactivation of the S protein [26]. It could also be explained by results found by Patrice et al., who demonstrated that virion particles replicating in epithelial cells of the respiratory tract in blood type A or B individuals are covered with A or B antigens, making anti-A and anti-B antibodies, present in sera from blood type O individuals, capable of specifically inhibiting the adhesion of SARS-CoV-2 S protein-expressing cells to ACE2-expressing cell lines [27]. The last study suggests that the absence of anti-A and anti-B antibodies in blood type AB individuals could generate a more severe form of COVID-19. In addition, several studies have demonstrated that non-O blood type individuals have a higher risk of having a positive test for SARS-CoV-2 [28]. In our population of pregnant women and newborns, this association was not addressed. In addition, we observed a discrete but significant difference in the mean age of mothers with a positive SARS-CoV-2 test who developed severe COVID-19 versus those who did not develop COVID-19 symptoms. A previous meta-analysis demonstrated the increased risk of suffering severe COVID-19 at every year of age [29]. The age difference observed in our study population was less than 3 years. Nevertheless, we wanted to address this point since age has been one of the more recognized risk factors in the general population. The data presented here suggest that specific studies in pregnant women are necessary for a better delineation of the effect of age during pregnancy. 

Furthermore, the relationship between ABO blood groups and cardiovascular diseases has been well established. Since 1976, the association of blood group A and AB individuals with a higher risk of developing thrombotic events has been described. Non-O blood groups have shown a higher incidence of both venous and arterial thromboembolic events, deep vein thrombosis (DVT), and pulmonary embolism (PE) [26]. Recent studies have shown that microthrombosis developing in the pulmonary vascular bed during COVID-19 infection seriously contributes to acute respiratory syndrome [30].

Pregnancy is considered a more susceptible state for viral infections because of immunological changes [31]. Physiological pregnancy adaptations may lead to lung vulnerability predisposing to a higher risk of hypoxemia and acute respiratory distress [32,33]. Another physiological adaptation during pregnancy and the peripartum period is a hypercoagulable state, which leads to increased levels of thrombotic factors such as VII, VIII, X, XII, von Willebrand factor, and fibrinogen and reduced levels of protein S and fibrinolysis [34]. According to the USA Centers for Disease Control and Prevention (CDC), pregnant women do not have an increased risk of death related to COVID-19 but may have a higher risk of hospitalization and mechanical ventilation than non-pregnant women [35]. Therefore, blood group AB in pregnant women with SARS-CoV-2 infection may lead to a more severe form of the disease. However, these results should be taken with caution since the number of AB individuals is small in our study population.

Based on the above reasons, it could be inferred that pregnant women also have a higher risk of death related to COVID-19, though, in the present study, we found no association with a specific blood type. This lack of association disagrees with other population studies, where an association of blood group A with a higher risk of death than blood group O has been established [36]. As pregnancy is a particular state where many cellular and molecular changes occur, we could not expect the same behavior as in the rest of the population. 

Blood group as a risk factor might not be extremely important in the general population. Still, as analyzed by Le Pendu et al., it should be considered a focus of interest in vulnerable groups, such as pregnant women [37]. Our results contribute to the knowledge regarding the risk factors implicated in SARS-CoV-2 infection and disease progression. The ultimate goal of the identification of the population at higher risk is the implementation of health policies to improve the quality of life of the population and the optimization of human and economic resources. 

This study contributes to the knowledge of risk factors in the Mexican population, which could have significant implications in different fields of prevention and management. Even in the vaccination era, COVID-19 will remain a public health issue since we must learn how to live with this virus. The cases of COVID-19 infection might be milder in the vaccinated population. Determining the risk factors that might be related to complications or susceptibility is key for reducing the impact and establishing protective measures. Our study has some limitations. First, the number of individuals in the AB group is small, less than 1% of the total study group. Nevertheless, the distribution of the blood groups in our study population is representative of the frequencies described for the Mexican population. These results need to be corroborated in other populations with a higher prevalence of the AB blood group. Second, the number of deaths included is relatively small and could compromise the identification of a true association with fatal outcomes. Gathering a larger number of affected individuals with a fatal outcome could reveal a discrete association. On the other hand, our study has some strengths. First, we analyzed a large number of individuals. Second, we used detection methods internationally validated, reducing the rate of false positive or false negative tests. 

## 5. Conclusions

Pregnancy is recognized as a more susceptible state for infectious and non-infectious diseases. Therefore, pregnant women deserve special attention to identify the main risk factors for acquiring COVID-19. This study did not find a higher risk of SARS-CoV-2 infection between blood groups in pregnant women. In addition, our results suggest that pregnant women with blood group AB are at higher risk for developing severe COVID-19 in the Mexican population. Therefore, they should have stricter vigilance from clinicians in our population to ensure a safe pregnancy course. Further studies, including larger samples in ours and other populations, are needed to reinforce our results.

## Figures and Tables

**Table 1:** Distribution of the study population according to blood groups.

Blood Group	SARS-CoV2 Test Negative		SARS-CoV2 Test Positive		*n* (%)
	Mothers	Newborns	Mothers	Newborns	
**O**	753	189	330	81	1353 (70.9)
**A**	208	57	96	24	385 (20.1)
**B**	92	19	32	7	150 (7.8)
**AB**	11	1	4	2	18 (0.9)
	1064	266	462	114	1906

**Table 2:** Association between blood groups and positive SARS-CoV-2 test.

Blood Group	*p* Value	OR (CI 95%)
**O**	Ref.	
**A**	0.616	1.384 (0.388–4.936)
**B**	0.530	1.509 (0.417–5.461)
**AB**	0.731	1.261 (0.337–4.726)

**Table 3:** Presence of severe symptoms among mothers by blood group.

Blood Group	Severe Disease	
	No	Yes
**O**	245	85
**A**	76	20
**B**	28	4
**AB ^a^**	1	3

^a^ Proportion comparison through the Pearson chi-square test revealed *p* = 0.029.

**Table 4:** Association of maternal death with blood groups among mothers with severe COVID-19.

Blood Group	Maternal Death		*p* Value	OR (CI 95%)
	No	Yes		
**O**	83	2	Ref.	
**A**	18	2	0.139	4.611 (0.609–34.939)
**B**	3	1	0.053	13.833 (0.965–198.262)
**AB**	3	0	0.999	0.000

## Data Availability

The data presented in this study are available on request from the corresponding author. The data are not publicly available due to it contains other clinical information not relevant for the present study.
